# Emerging prospects of macro- and microalgae as prebiotic

**DOI:** 10.1186/s12934-021-01601-7

**Published:** 2021-06-05

**Authors:** Anil Kumar Patel, Reeta Rani Singhania, Mukesh Kumar Awasthi, Sunita Varjani, Shashi Kant Bhatia, Mei-Ling Tsai, Shu-Ling Hsieh, Chiu-Wen Chen, Cheng-Di Dong

**Affiliations:** 1 Department of Marine Environmental Engineering, National Kaohsiung University of Science and Technology, Kaohsiung City, 81157 Taiwan; 2 College of Natural Resources and Environment, Northwest A&F University, Yangling, Shaanxi Province 712100 People’s Republic of China; 3 Gujarat Pollution Control Board, Gandhinagar, Gujarat 382010 India; 4 Department of Biological Engineering, College of Engineering, Konkuk University, Seoul, 05029 Republic of Korea; 5 Department of Seafood Science, National Kaohsiung University of Science and Technology, Kaohsiung, 81157 Taiwan

**Keywords:** Microalgae, Macroalgae, Seaweeds, Dietary fibre, Prebiotics, Polysaccharides

## Abstract

Macro- and microalgae-based foods are becoming popular due to their high nutritious value. The algal biomass is enriched with polysaccharides, protein, polyunsaturated fatty acids, carotenoids, vitamins and minerals. However, the most promising fraction is polysaccharides (PS) or their derivatives (as dietary fibers) which are not entirely fermented by colonic bacteria hence act as potential prebiotic. Primarily, algae become famous as prominent protein sources. Recently, these are widely adopted as functional food (e.g., desserts, dairy products, oil-derivatives, pastas etc.) or animal feed (for poultry, cattle, fish etc.). Besides prebiotic and balanced amino acids source, algae derived compounds implied as therapeutics due to comprising bioactive properties to elicit immunomodulatory, antioxidative, anticancerous, anticoagulant, hepato-protective, and antihypertensive responses. Despite the above potentials, broader research determinations are inevitable to explore these algal compounds until microalgae become a business reality for broader and specific applications in all health domains. However, scale up of algal bioprocess remains a major challenge until commercial affordability is accomplished which can be possible by discovering their hidden potentials and increasing their value and application prospects. This review provides an overview of the significance of algae consumption for several health benefits in humans and animals mainly as prebiotics, however their functional food and animal feed potential are briefly covered. Moreover, their potential to develop an algal-based food industry to meet the people's requirements not only as a sustainable food solution with several health benefits but also as therapeutics is inevitable.

## Background

Recently, there has been a growing interest in functional foods as well as the prebiotic potential of foods for numerous health benefits [1–3]. Functional food can provide not only the nutrition but also the positive health effects against numerous conjoint diseases appearing in recent times. It can be proactive against those diseases and must carry properties like anti-inflammatory, antioxidant, antimicrobial, and antiviral, moreover be preventive for constipation, gastric ulcers, diabetes, anaemia, and hypertension. However, prebiotic potential is such a unique characteristic of certain foods which hardly get digested in the host’s gastrointestinal tract or fermented by the host’s gut microbiota. Therefore, it helps to enhance growth of health beneficial organisms called probiotics in the lower gastrointestinal tract or colon.

Apart from functional foods, several foods with and without prebiotic potentials are also blended with probiotics for improving their positive health effects. There are a large number of probiotics existing in numerous dairy products to improve gut health for example yoghurt, curd, cheese, and ice-cream. They are comprising a diverse group of health-boosting microorganisms. In which some are usual dwellers of the gut and some as fermentative bacteria. The latter are utilised in the food industries for improving processes and product quality, *e.g.,* texture, flavour and stability. They have specialized enzymes and mechanisms to perform such effects precisely in adverse gut conditions [4, 5]. Several group of probiotic bacteria such as *Bifidobacterium, Lactobacillus, Bacillus, Streptococcus, Saccharomyces* and *Lactococcus* have been investigated and most of them are certified by health organizations in food products due to their specific positive health effects [4, 6–9]. These probiotics are recognized for numerous health effects including immunity enhancement, diarrhoea prevention, constipation inhibition, [10, 11], lactose intolerance, blood cholesterol reduction and cancer prevention [12] and associated side effects [1]. Moreover, probiotics also protects against several opportunistic pathogens [13].

In recent years, evidence has appeared for the positive health effects of foods, food ingredients or biochemical compounds derived from certain macro- and microalgae. These algae potentially show the widest range of products of the microbial world owing to their nutritional quality [14], in which some are important sources of human and animal foods [10, 15]. Some compounds exhibit the prebiotic potential to support probiotic growth in the host gut upon consumption [16]. Microalgal biomass comprised of a wide range of bioactive compounds such as protein, polysaccharides, pigments, vitamins, polyunsaturated fatty acids (PUFAs), and minerals as intracellular compounds and oligosaccharides as extracellular compounds [17–19]. Among them, the most promising found to be polysaccharides (PS) and their derivatives (as soluble fibres). Some of these PS (*e.g.,* exopolysaccharides, fucoidans, alginates, and carrageenans) are not fermented completely by colonic microbiota and act as prebiotic. However, growth promotion and performance of probiotic by prebiotic microalgae is not limiting by these compounds directly, such enhancements are also reported indirectly such as suppression of pathogens, removing toxic substances, improving gut adsorption, improving disease resistance and immunity, enhancing their viability and storage etc. which are summarized in Table 1. Researchers were also investigating health improving bioactive compounds as well as whole dried biomass of macro- and microalgae. Their attributes greatly depend on composition of the biomass as well as on the species and growth condition provided. Scientific evidence is still lacking about probiotic roles of microalgae in humans, though intermittent studies have exhibited the probiotic role in marine animals.

**Table 1:** Prebiotic role of various algae strains on growth promotion of probiotics and related health improvements

Microalgae sp.	Probiotic	Microalgae conc (mg. ml^−1^)	Main focus Of study	Other remarks	Reference
*Chlorella vulgaris*	*Lactobacillus brevis*	0.1–1.5%	Improving the probiotic growth, health, product yield and other desirable properties	Algae shortening the log phase, improving lactic acid yield, enzyme activity and acidifying activity of probiotics	[20]
*Euglena gracilis*	*Streptococcus iniae*	ND	Development as animal feed, paramylon activity was tested	Immunostimulant activity offered to the animal host	[21]
*Pavlova pinguis*	*Phaeobacter inhibens*	*ND*	*Disease management in bivalve V*. *coralliilyticus*	Vibrio sp. infection reduction for reducing the mortality of larval shellfish	[22]
*Chlorella vulgaris* and *Spirulina platensis*	Lactic acid bacteria	3	Supplementing microalgae in milk products for improving its storage and self-life	Increasing the viability of probiotics in final product but also the sensory attributes	[23]
*Euglena gracilis*	*Bacillus licheniformis* *or B. subtilis*	ND	Development as animal feed, β-glucan was tested in poultry, cow, horses, dogs, cats, birds and reptiles	Improved the health and immune system of animal hosts	[24]
*Spirulina platensis*	*Lactococcus lactis sp.*	1	Supplementing microalgae in yogurt to improve health benefits due to probiotic enrichment	Increasing the viability of probiotics and lactic acid bacteria	[25]
*Spirulina platensis*	*Lactococcus lactis sp.*	1	Supplementing microalgae in yogurt to improve health benefits due to healthy bacterial enrichment	Increasing the viability of probiotics and lactic acid bacteria	[26]
*Phaeodactylum triconutum, Tetraselmis chuii*	*Bacillus subtilis*	ND	Developed as animal feed, effect of protein fraction was examined	Immune system was improved and intestinal adsorption was increased	[27]
*Spirulina platensis*	*Bifidobacterium bifidum and other*	1–2	Feed for animals suffering from disease due to imbalance of insulin and adipose distributions	It helped to adsorb metal ions in animal gut to restore gut disorders	[28]
*Spirulina platensis*	*Lactobacillus acidophilus, L. Casei, S. thermophilus*	5–10	Stimulating growth of lactic acid bacteria	Three LAB have been improved in their viability and activity, and suppressed the growth of pathogenic bacteria, improved intestinal adsorption of host	[29]
*Dunaliella tertiolecta*	*Bacillus sp.*	ND	Development as animal feed, β carotene effect was tested in shrimp	Improved immune system and disease resistance	[30]
*Spirulina platensis,* Chlorococcum, D. salina, S. magnus, Chlorella	*Lactobacillus lactis, Lactobacillus bulgaricus and Bifidobacterium longum*		Stimulating growth of lactic acid bacteria	Xylose and galactose in algal extract stimulate the growth of probiotics	[31]
*Navicula sp.*	*Lactobacillus sakei*	ND	Developed as animal feed, Oligosaccharide effect was tested	Immune system was improved and antioxidant property was enhanced	[32]

*ND* Not determined

Algae are multicellular, eukaryotic, non-flowering, photosynthetic aquatic plants which include microalgae, macroalgae (seaweeds) and sometimes unicellular cyanobacteria. They are constituting the base of aquatic food chains. Phylogenetically they are distinct and encompassing different phyla and classes [10, 33, 34]. These algae grow well in all types of aquatic environments, for example freshwater, marine, and hypersaline, also moist soils and rocks [35]. They are recognised for several potential applications such as functional food [16, 36, 37], animal feed [16, 21], biomedicals [38, 39], prebiotics [33, 40, 41], cosmetics [42], and organic manures [43], wastewater treatment [43–45] high value [46] and biofuel production [15, 47]. Furthermore, several studies have addressed health benefits of such microalgal compounds comprising antioxidant, anti-inflammatory, antimicrobial, antiobesity, and anticancer properties, besides hypocholesterolemic characteristics. Thus, it serves as nutraceuticals [38, 39]. The demand in algae-based food and feed ingredients in the food market is expected to grow soon; however, steady applications exist mainly in the aquaculture and dairy industry [48]. Moreover, an existing trend has been marked to blend microalgal biomasses into fermented milks to improve the medicinal and nourishing attributes via promoting the probiotics stability [49, 50]. Table 2 summarizing the challenges and their possible solutions for microalgae probiotic formulations in milk products to enhance their commercial attributes and applications. Nevertheless, before seeking application of algal-based products, it is important that microalgae cultivation and related facilities must be cost-effective.

**Table 2:** A summary of prebiotic microalgae formulation in milk products for technological improvements

Technological attributes and remunerations of microalgae supplementation	
Challenges	Probable solutions
Increasing cost of final product	Cost-effective production of microalgae added healthy fermented milks
It leads to sensory flaws due to oxidation of unsaturated fatty acids	Add fruit flavors (kiwi, strawberry) to suppress off flavor of microalgae addition
Lower product texture and color options due to non-solubility of microalgal powders	Improving product texture and color range by external green sources by homogenizing them effectively
**Product property**	
Lower viability of healthy bacteria in milk products due to lower prebiotic effects and high active oxygen sp.	Improving their viability by microalgal prebiotic effects: altering redox potential, improving O_2_ scavengers (vit. C, β-carotene, carotenoids) and nutritional level (amino acids, minerals, peptides, B-vit etc.)

In the recent development on algae cultivation, they are not limited to only photoautotrophic cultivation mode, under which they only can utilize inorganic carbon (CO_2_) and not to organic carbon to enhance their growth using dual pathway photosynthesis and oxidative phosphorylation. Thus, a new cultivation strategy of microalgae to grow them mixotrophically is very important to remove the economic constraints and their effective exploitation for obtaining higher biomass [51–53]. Moreover, another advantage as in the CCU technology, algae platform is most promising among others specially for increased CO_2_ mitigation rate mainly due to their higher productivity than any other plants [15, 54]. These attributes along with mixotrophic cultivation mode can greatly reduce challenges associated with their biomass harvesting, shelf life extension and constrained industrial viability. Recent advances in microalgae research could be a breakthrough towards exploiting high throughput screening techniques to sort out potential strains, especially high yielding desired products for health applications [55].

The main aim of this short review is to highlight recent research developments on widening applications of algae-based products in functional foods, animal feed, nutraceutics and/or therapeutics, encompassing products of macro- microalgae/cyanobacteria, which independently or with some formulations exhibit potential to improve human and/or animal health. The knowledge gaps between research and development as well as stage of commercialization of these products are also discussed briefly.

## Prebiotic research advancements

### Prebiotic concept improvements

Usually, prebiotics are assumed to offer a selective effect on the host microbiota which leads to their improved health. When prebiotics are not well fermented, they often exert an osmotic response in the host GIT, whereas once they are effectively fermented by GIT flora shows higher metabolic gas production and exert its prebiotic effect [56].

Prebiotics works as growth stimulators to commensal bacilli such as *Lactobacillus sp.* These are known bacteria for improving GIT barrier function during external stress by protecting the tight epithelial junction [57]. By observation, approved prebiotics mainly augment the count of *Bifidobacteria* in the human GIT [58]. The general finding suggests that the above benefit in the human health offered by pathogens removal as well as immune system modulation [59]. *Bifidobacteria* can metabolize carbohydrates having shorter chain lengths and known as oligosaccharides [60]. Study shows that these prebiotics can modulate the gut microbiota especially promoting *Bifidobacterium* group [61]. For this, prebiotics not only alter the mucosal lining of the colon but also the transportation of the SCFAs across trans-epithelium. In which, transportation of cationic minerals is induced by the reduced abdomen pH.

Prebiotics can be served as a substitute to probiotics or as a supplementary boost for them. longer stability of prebiotic, durability during processing, and their physicochemical characteristics can encourage prebiotics compared to probiotics [62, 63]. Also, high tolerance to gastric acids, bile salts, and hydrolytic proteases occurring in GIT could be other desirable attributes of prebiotics. Moreover, prebiotics are leading to lower intestinal pH and promote osmotic water retention in the bowel [64]. However, it was recorded that excessive prebiotics intake can cause diarrhea and abdominal gas. Instead, prebiotics at an optimum amount exert several positive health effects and override all adverse effects. Prebiotics are not allergenic compounds, also not proliferating the genes involved in antibiotic resistance. Although the impact of pathogens removal by prebiotics could be less than antibiotics, their desirable attributes discussed above to support them as a natural potential alternative for antibiotics [64].

**Fig. 1:**
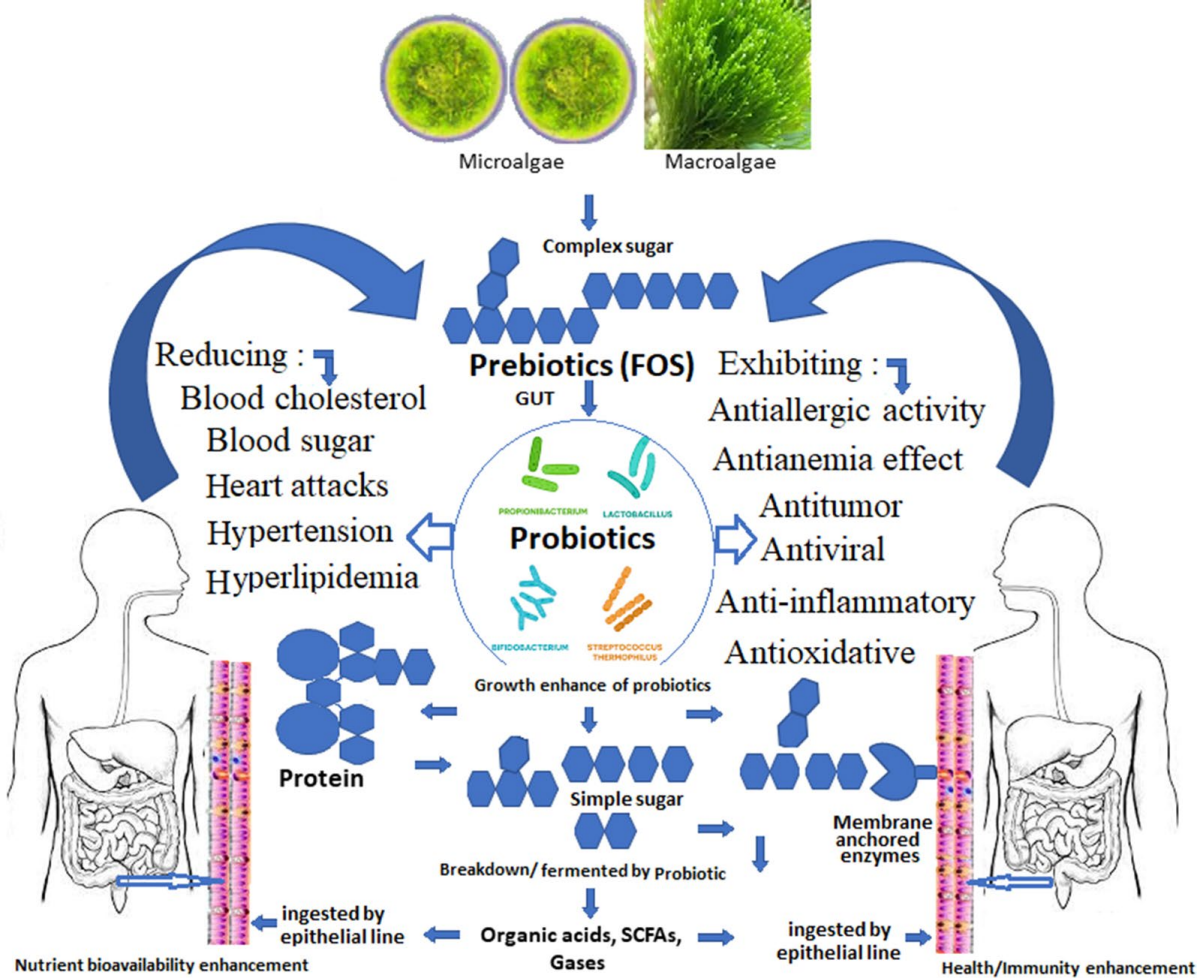
Overview of prebiotic digestion process via probiotic in human gut and associated health benefits

Primary definitions were revised with times as per the development appeared on novel prebiotics and understanding up on their structure and metabolic mechanisms with respect to gut flora (Fig. 1). Prospects of the specified prebiotics effect was extended by International Scientific Association for Probiotics and Prebiotics in 2003, and defined that prebiotic effects were not limited to the colon, it also reaches to the skin, mouth, abdomen, intestine, and vagina [65]. In this, during the prebiotic convention in the year 2008, the most important modifications in the prebiotic’s phenomenon taken place by the Food and Agriculture Organization [66], where prebiotics were characterized “a nonviable edible which exert several health benefits to the host via alteration of the microflora.” Such description deleted the measures of specificity and limitation to the GIT. Moreover, extended the lists of prebiotics beyond FOS, inulin, HMO, GOS, and lactulose. Hence, novel prebiotics have been included for example resistant starch, sugar alcohols, XOS, SOS, lactosucrose, IMO, and POS. Accordingly, the necessity of only GIT flora has been removed to metabolize the prebiotics, authors also recommended the exclusion of selectivity obligations. Moreover, this classification underlines the prebiotics-based ecological and operational characteristics of the GIT flora, for example ecosystem diversity, also a mixed microbiota and the SCFAs production [67].

Regardless of the above refinements, experts, strongly demanded for specificity correlative with taxonomic groups or positive metabolic functionalities must remain the main criteria for prebiotic selection and classification [68, 69]. This amendment shows, prebiotics may not be completely metabolized, instead digested by precise microbes in a way promoting the health of the host. However, the selectivity perseverance would not ignore the impacts on species which are not dominant like *Bifidobacterium and Lactobacillus*. For example, some prebiotics found to encourage the growth of butyrate producing *Firmicutes sp*. They are advantageous to colonic health [60]. Whereas *Bifidobacterium sp.* are not a producer of butyrate.

Towards a new amendment in prebiotic development, *Clostridium leptum*, *Faecalibacterium prausnitzi*, *Akkermansia muciniphila,* and *Bacteroides fragilis* are known probiotics exerting positive effect against obesity and colitis, In which, *Clostridium* and *Bacteroides* groups are also involved to produce some health detrimental toxic metabolites, In this context, the recent ISAPP agreement panel now recommends new prebiotic definition: “any substrate which specifically uptakes by the host to exert a health effect” [60].

### Role of prebiotics in intestinal microflora

The impacts of ingested prebiotics on human GIT microbiota are well addressed. These prebiotics have a major role to alter the abundance of certain microorganisms after a few weeks of their consumption based on their compositions and structures [16, 18]. In previous studies, the incidence of *Bifidobacterium* augmented in two weeks period with 15 g.d^−1^ oligofructose or inulin ingestion, and reduced the density of *Clostridium, Bacteroides,* and *Fusobacterium* from oligofructose and gram-positive cocci from inulin [70]. Other classical prebiotics such as FOS and GOS, have exhibited the great abundance of actinobacteria improved substantially with prebiotics dosing, which are primarily known to induce *Bifidobacterium* population. Majority of the studies discovered the growth augmentation of *Bifidobacterium* followed by *Lactobacillus* by these prebiotics consumption, other studies also described increase the numbers of F*aecalibacterium* and *Atopobium sp* [71]. The count of GIT bacteria also reported to reduce after the ingestion of these prebiotics, maybe due to competition with other species which specially ferment the same prebiotics in the human intestine. Nevertheless, fatty acids, mainly SCFAs, which are intermediate of prebiotic metabolism, found to encourage variations in the GIT microbiome, includes colonic pH reduction which also inhibits many bacteria such as *Clostridium* and *Bacteroides* [71].

Overall, these studies revealed that prebiotics certainly have potential to modify gut environments for advantageous members while reducing chances to proliferate harmful bacteria in the GIT environment and progressing the composition of colonic microbiota of the host towards healthier. However, still the consensus has not been set about which microbes are positive or negative members of the gut [67]. These shortcomings suggest more studies for establishing a comprehensive association between prebiotics and GIT microbiota.

Recently, with the progress in prebiotics research and associated GIT microbiome range *e.g., Eubacterium, Bacteroides, Roseburia, Faecalibacterium, Akkermansia* and *Ruminococcus* have been main targets of prebiotics [72]. A human trial showed, the FOS intake stimulated the abundance of butyrate-synthesizing microorganisms such as *Ruminococcus, Faecalibacterium* and *Oscillospira* which are detected in the feces [73]. Previous studies [74, 75] addressed that seaweed dietary polysaccharides could augment the count of *Bacteroides* in mice feces and their fundamental mechanism attributed the specific PULs expedite its absolute metabolic niche. Similarly, count of *Faecalibacterium* in the healthy adult feces significantly rose in the 16-d period after 10 g d^−1^ inulin ingestion [76]. A 3-month treatment of obese women with 16 g d^−1^ dietary inulin-type fructans resulted an enhancement in *Faecalibacterium* [77]. An 8-week in vivo study demonstrated a 10-time rise in the count of F*aecalibacterium* in the feces of an adult with the consumption of 1-kestose at 5 g d^−1^ [72]. Oligosaccharides obtained from lemon waste augmented *Faecalibacterium, Roseburia* and *Enterobacter* recorded by in vitro study carried out with feces inocula [78]. The *Akkermansia* count in mice feces was enhanced over 100-fold with the FOS ingestion [79]. Likewise, uptake of polyphenols rich fruits, mainly grapes, also enhanced the *Akkermansia* count [80].

### Prebiotic mechanism of action in GIT condition

Prebiotics are partly metabolized in the higher sections of the gastrointestinal tract as the human genes do not transcribe certain carbohydrate hydrolysing enzymes called CAZymes [81]. When prebiotics compounds (FOS, GOS, inulin, and lactulose) and dietary carbohydrates (XOS, PDX, SOS, resilient starch, gluco-oligosaccharides, lactosucrose, etc.) with recognized prebiotic effects arrive in the colon then they are specifically fermented by hydrolytic microbes [82]. This process produces several metabolites such as organic acids (lactate, succinate and pyruvate) short chain fatty acids (C1–C4), and gases (CO_2_, H_2_, CH_4_, and H_2_S) which help in the intestinal metabolic balance, leading to the reduction in nitrogen-based final products, colonic pH, and faecal enzymes [83]. The above prebiotic specificity for intestinal bacteria is separated into two groups (I) lactate and acetate fermentative (*Bifidobacterium and Lactobacillus sp*) and lactate and acetate consumers (*Eubacterium, Faecalibacterium* and *Roseburia sp*) for improving butyrate formation. It can be concluded that there are two routes, one is direct growth stimulation of these intestinal bacteria by consuming prebiotics and second is growth stimulation of other gut microbiota from their metabolites such as acetate.

Several studies have demonstrated how these prebiotics exhibit precise health effects upon its consumption which have been recorded case by case. For example, to improve bowel condition and colon condition in patients of IBD, IBS, Ulcerative colitis, Crohn’s disease etc. Prebiotic dosing reduces the pro-inflammatory immune markers and improves the calprotectin performance. It also enhances the cytokine production. Prebiotic effectively reduces the IBD symptoms by modulating the *Bifidobacterium* counts upon is appropriate dosing and much enhancement was observed in butyrate supplemented systems [69, 84–86]. For improving GIT condition from colon cancer, prebiotics usually show substantial reduction in the number of putrefactive compounds generation by colonic microflora from butyrate, especially *Bifidobacteria* play a major role to down regulate the carcinogenic promoters as well as reduce the genotoxins level on biomarkers which is leading to cell proliferation with reduced cancer features [86, 87]. Prebiotic found to improve bone mass and density by enhancing calcium absorption and through reducing GIT pH due to production of SCFAs [69, 85]. Mechanisms to regulate the gut metabolism and digestate transit with the reduction in onset of constipation, dysentery and diarrhea. To improve the host heath from antibiotic-linked and traveller-diarrhoea, prebiotics exhibit functionality to reduce the fever and vomiting in children through inducing the growth of *Bifidobacteria*. It was also observed that probiotics can reduce the prevalence of diarrhoea upon regular optimized intake [86, 88]. The mechanisms of prebiotics, for improvement in the host immune system has been described through the production of pro-inflammatory cytokines (TNF-α) and by stimulating overexpression of receptors on macrophages and lymphocytes B and T cells [69, 89].

From several recent studies, carried out in vitro and in vivo revealed that the gut flora apparently plays a much additional key role for the host’s health than it was formerly apprehended, and this microbiota can be selectively modified by various important groups of prebiotics. Among all, a various polysaccharide groups can elicit their effect through various noticeable mechanisms such as (a) specific fermentation (b) the pH of the GIT (iii) bulking of fecal matter (iv) pathogens inhibition for gut colonization (v) prevention of putrefactive bacteria to avoid toxic metabolites production for the host. Algal Oligo- and polysaccharides could exhibit health effects similar or more effective than the products derived from other sources. This is obvious through biochemical characterizations, especially for some oligo- and polysaccharides from marine macro- and microalgae which are undigested by human enzymes in the upper region of the GIT. Thus, these algal Oligo- and polysaccharides offer a great potential as an emerging prebiotic for health application, especially for microalgae, it is more opportunities to develop a cost-effective biorefining process for extracting these products from harvested wet algal biomass or dried biomass as such or as nutraceuticals [90]. They can be encompassed as human food, animal feed, and/or administered as liquid drinks and solid/semisolid pills Moreover, the advances of novel enzyme technologies especially from marine, algae, bacteria and molluscs will enable us to explore these marine PS towards developing novel prebiotics regimen. Table 3 summarizing the name of prebiotic microalgae with their specific bioactive compounds responsible for various health benefits upon precise applications.

**Table 3:** Prebiotic algae with specific bioactive compounds exhibiting various health benefits upon precise applications

Macro/microalgae	Commercial biomass form	Products	Bioactive compounds	Positive health effects	Reference
*Chlorella sp., Arthrospira platensis*	Powder	Cheese	Carbohydrates, protein, ω3-FA	Anticancer; lowering gastric ulcers, neurosis, hypertension, anemia, constipation, diabetes, infant malnutrition,	[91]
*Spirulina sp.*	Powder and extract	Non-alcohol beverage	Protein, chlorophylls, phycocyanin	Enhanced immunity and lymphatic performance, anticancer and antiulcer property	[92]
*Tetraselmis suecica*	Food supplement	Extract	–	Prevention from diabetes and obesity	[93]
*Hematococcus pluvialis Phaeodactylum* *tricornutum*	Powder or flour	Biscuits	Protein, ω3-FA, DHA, EPA, astaxanthin	Antioxidative response	[94, 95]
*Chlorella sp.* *Schizochytrium sp. Thraustochytrium sp.*	Food supplement	Powder, flour, tablet or liquid	Proteins, ω3-FA	Prevent from constipation, satiety induction	[96]
*Ulva, Porphyra, Laminaria/saccharina, Enteromorpha, Undaria, Rhodella, Fucus, Ascophyllum, Sargassum*	Food supplements	Powder	Polysaccharide	Immunomodulatory, Antilipidaemic and hypocholesterolaemic	[39, 97]
*Dunaliella sp. Spirulina sp.*	Powder	Miso	Protein, vitamins, minerals	Antioxidative response	[98]
*Arthrospira platensis*	Oil	–	Carotenoids	Antimicrobial and antiviral properties	[99]
*Dunaliella salina*	Culinary condiment with sea salt	Powder	Carotenoids	Antioxidative response	[100]
*Porphyridium*	Food supplements	Powder	Polysaccharide	Immunomodulatory, Antilipidaemic and hypocholesterolaemic	[39, 97]
*Arthrospira platensis, Chlorella sp.*	Powder or flour	Bread and cookies	Protein, vitamins, minerals	Reduction in cholesterol and fat levels, satiety induction	[101]
*Gracilaria, Cladosiphon, Monostroma, Capsosiphon, Kappaphycus, Furcellaria, Soliera*	Food supplements	Powder	Polysaccharide	Immunomodulatory	[39, 97]
Haematococcus pluvialis	Food supplement	Capsules	Astaxanthin	UV protection, anticoagulatory & anti-inflammatory effects, immunity modulation, improve cardiovascular health	[102]
*Chlorella sp. and Spirulina sp.*	Powder and extract	Milk	Proteins, ω-3FA, EPA, DHA	Reduced onset of anemia	[103]
*Chlorella, Phaedactlylum, Gyrodinium,*	Food supplements	Powder	Polysaccharide	Immunomodulatory	[39, 97]

## Prebiotic potential of algal compounds

Prebiotics potential were observed in some compounds of seaweeds and marine microalgae, mainly native as well as modified forms of polysaccharides (PS) were recognized as prebiotics such as XOS, GOS, AGAROS, ALGOS, NAOS, galactans, arabinoxylans, β-glucans. These algal PS are usually not digested by metabolic enzymes in the upper gut. Therefore, they can be used as dietary prebiotics and able to augment the growth of probiotics [104]. Specific PS found in certain algal biomass having probiotic potential have been described with their monosaccharide compositions and the linkage types, moreover some di- and oligosaccharides which are part of the PS of some microalgae are also described as fibers. Fucoidans: Brown seaweeds are rich in fucoidans, a soluble homo- or heteropolymeric PS, in which L-fucose are the main sugar residue. It is an irregularly branched and sulphated high molecular weight polysaccharide (HMW-PS), whose monomers are linked by alternating (1,3)- and (1,4)-α bonds. Galactofucans are another PS found in *Laminaria* and *Undaria* brown macroalgae [39].

Alginates are major approx. 20–29% DW carbohydrates in *Fucus, Ascophyllum* and *Sargassum*. These species also contain fucoidans in lower amounts (10–11% DW) [105]. It is an anionic-acidic, water soluble, non-branched PS, being used in the food industry (E400–E407), it comprises L-guluronic acid and D-mannuronic acid monomers. Alginates mainly occur in both *Laminaria* and *Macrocystis*. A β-glucan for example Laminaran, (1,3)-and (1,6)-β-linkages with some other laterally linked sugar residues found in *Laminaria, Ascophyllum, Undaria* and *Fucus*. [106]. *Carrageenans* are broadly used as gelling agents in the food industry. Moreover, polysaccharides reported from green seaweeds includes: ulvan as main PS in *Enteromorpha* and *Ulva* species, Capsosiphon (1,3-β-mannan) in *Codium fragile*, Rhamnans in *Enteromorpha*, galactans in *Caulerpa species* and other PS have also reported [39].

On the other hand, there are not many reports over complex PS from microalgae, except β-glucan and homogalactan respectively in *C. vulgaris* and *Gyrodinium*, other PS are usually heteropolymers comprising numerous different monosaccharides. The glycosidic linkages of these PSs were poorly described for limited PS, for example PS from *Phaeodactylum tricornutum* and *Aphanothece halophytica*. But the simple polymeric structures especially for replicating mono-, di- and oligosaccharides were well explained for several PSs from *Porphyrium, Arthrospira* and *Rhodella* [39]. Hemicelluloses (HC) are most common soluble PS in the algal biomass, HC are branched polymers found in the cell as well as produced/released into the culture medium. HC are heteropolymers and can be simply hydrolysed by hemicellulases as well as by acid and basic solutions. Moreover, PS, which are non-soluble fibers for example cellulose found in seaweeds, is a non-branched linear polymer composed of mainly anhydrous glucose residues which are linked together by β-(1,4) linkages. Lignin is also a non-soluble fiber, which is resistant to microbial enzymes [85]. Table 4 summarizing the dietary fibers from macro- and microalgae sources reported for promotion of specific probiotics and suppression of other harmful gut bacteria.

**Table 4:** Various prebiotics recorded for affecting probiotic abundance in GIT environment

Prebiotic components	Induced bacteria	Suppressed bacteria	Reference
FOS	*Lactobacillus, Bifidobacteria,* *Ruminococcus, Faecalibacterium,* *Oscillospira*	–	[73]
Fractan*	*Bifidobacteria, Anaerostipes*	*Bilophila*	[107]
GOS	*Bifidobacteria*	*Holdemania, Synergistes* *Dehalobacterium, Ruminococcus,*	[108]
Inulin	*Actinobacteria*	*Clostridia*	[109]
GOS	*Bifidobacteria, Bacteroides,* *Atopobium*	*–*	[110]
Inulin (long chain)	*Lactobacillus, Bifidobacteria,* *Atopobium,*	*Bacteroides-Prevotella*	[111]
Fractan*	*Bifidobacteria, Lactobacillus*	*–*	[112]
FOS	*Bifidobacterium*	*Salmonella, Phascolarctobacterium Enterobacter, Coprococcus, Turicibacter*	[108]
-NAOS	*Bifidobacteria, Lactobacillus*	*Bacteroides, Enterococci,* *Putrefactive bacteria*	[113]
Alginate	*Bifidobacteria, Lactobacillus*		[114]
Fucoidan	*Lactobacteria*	*–*	[115]
Fractan*	*Bifidobacteria, Faecalibacterium prausnitzii*	*Bacteroides, Propionibacterium*	[77]
Oligos/PS in Ascophyllum biomass	*Lactobacillus, E. coli*	–	[116]
GOS	*Bifidobacteria, Actinobaceria*	*Bacteroides*	[117]
Oligo/PS in Gelidium extract	*Bifidobacteria*	–	[118]
Agave inulin	*Actinobacteria, Bifidobacterium*	*Lachnobacterium, Desulfovibrio* *Ruminococcus*	[119]
Resistant starch Type 4	*Bifidobacteria, Parabacteroides distasonis, Clostridia*	–	[120]
Oligo/PS in Spirulina biomass	*Bifidobacterium, L. casei, L. acidophilus, S. thermophillus*	*P. vulgaris, B. subtilis, B. pumulis*	[29, 121]
Oligo/PS in Isochrysis biomass	*Lactic acid bacteria*	–	[122]

^*^Fractan: matching Inulin structure

In addition to PS, several other important bioactive compounds produced by algae which are showing comparatively fewer prebiotic properties and have reported several health benefits. As mentioned in the previous section, microalgae are a promising source of these compounds like proteins, steroids, carotenoids, fatty acids, lectins, minerals, vitamins, amino acids, halogenated compounds, and polyketides [123]. Microalgae produce essential amino acids, minerals, unsaturated fatty acids, and several vitamins (A, B, E, and K) and serve as functional foods for therapeutic and nutraceutical applications [95, 124, 125] which are well described in Table 5. Prebiotics commonly oblige as substrate to be biologically degraded by the colonic microflora with the help of enzymes. These prebiotics can be oligosaccharides, dietary fibers (mainly PS having DP > 10), resistant starches, sugar alcohols, non-absorbable sugars, proteins, amino acids, and also could be other biomaterials, such as mucins, microbial metabolites and products obtained from cell lysis. From recent studies it was understood that both macro- and microalgae are promising sources of the majority of above compounds, few of them are already verified to possess prebiotic attributes [69].

**Table 5:** Use of microalgae products and their specific applications in various health sectors

Category	Microalgae used	Products	Nutrient source/Health effects
Nutraceutics	*Spirulina, Nannochloropsis, Dunaliella, Schizochytrium* etc	Fatty acids and sterols, fibres, carbohydrates (EPA, DHA, GLA, SDA, Poriferasterol, Clinosterols, agar, alginates etc.) Carotenoids (β-carotene, astaxanthin, lutein, fucoxanthin etc.) Protein and amino acids (Single Cell Protein-spirulina, phycocyanin) Vitamins and minerals	High Saturated/unsaturated fatty acids, high fibers, high carbohydrates for nutrition Antioxidative effects Enriched with essential amino acids and good protein source Source of high vitamins (A, B2, B6, B8, B12, E, K), high minerals (Fe & Ca)
Therapeutics and/or Pharmaceutics	Genetically modified microalgae strain such as *C. reinhardtii, Schizochytrium, Spirulina, Chlorococcum, Haematococcus, Chlorella* etc	Specific microalgal extract, lotions (enriching either bioactive compounds, Tyrosine inhibitors or hydrolytic enzymes, Phytases, etc.)	Reducing blood cholesterol, antiallergic activity, decreasing blood sugar, antianemia effect, reducing heart attacks, antitumor activity, antiviral activity, reducing hypertension, reducing hyperlipidemia, improving immunity, stress reducing action, protecting from harmful chemicals, anti-inflammatory and antioxidative activities against neurodegenerative disorders, atherosclerosis disorders, T2DM, Cancer etc
Cosmeceutics	*Spirulina, Haematococcus, Dunaliella, Chlorella *etc	Moisturizers and lotions: Antiaging and UV-protection Polysaccharides Antioxidant enzymes Microsporin like amino acid Skin whitening and haircare	Poly unsaturated fatty acids (PUFAs) Carotenoids (astaxanthin, fucoxanthin) Fucoidan, alginates, galactans, agar, ulvans etc Superoxide dismutase, catalase, peroxidases, and glutathione Fucoxanthin, microalgal extract

Nevertheless, the health benefits determined in vitro or in vivo studies, more human trials must be completed to establish the optimized doses and the health effects in hosts for attesting positively confirmed prebiotic candidates to be approved finally for human use. For launching prebiotics from macro- and microalgae, such more trials are yet to be done especially for most of those PS obtained largely from them and to be recognized as safe prebiotics. Moreover, already established oligo- and polysaccharides (XOS, GOS, galactans, xyloarabinans, β-glucans), as prebiotics from these algae must be effectively outlined [126]. According to a past review, specific characters were not well-focused for screening of the potential prebiotics, therefore recent past PS from macro- and microalgae have already been subjected to human trials [39].

## Microalgae models and their products in use as nutraceutics/therapeutics

Various microalgal strains and their derived compounds have been already approved which have to be used either as food or food additives in various countries. For example, microalgae *Tetraselmis chuii* and *Ulkenia sp.* have been approved in Europe [127, 128]. *Arthrospira sp.* approved in the United States with the GRAS Notice [129], whereas its product phycocyanin is approved as a food additive only in Japan [130]. *Euglena gracilis* is approved as food in the United States recently [131]. DHA rich oil from *Schizochytrium sp.* has been approved as food in Europe through three Commission implementing decisions [132–134] and in Australia, New Zealand and Japan with a GRAS Notice and Schedule 25 respectively [135, 136]. However, DHA rich oil from *Ulkenia sp*. is approved to use in the United States, Australia and New Zealand with a GRAS Notice and Schedule 25 respectively [136, 137]. DHA rich oil from other microalgae species are approved only in the United States such as *Dunaliella salina, Auxenochlorella protothecoides* and *Chlorella vulgaris* with independent GRAS Notices [138–140]. DHA rich oil from *Chlorella sp.* are approved for human consumption as well as Carotene from *Dunaliella sp.* and *Haematococcus* algae color are also approved to be used as food additives in Japan [130].

The PS discussed in the above section for probiotic growth promotion, however its biodegradability and bioconversion are also tied to the huge variability of activities they encompass, which make them a promising material as pharmaceutics, therapeutics, and regenerative medicine [90]. Number of desirable activities were confirmed in PS and their derivatives both in vitro and in vivo, such as immunomodulatory, anticoagulant, antithrombotic, antitumor and anticancer activities. Moreover, they also found promising antilipidemic and hypoglycemic, antioxidants, anti-inflammatory and antibiotics agents. Other medicinal characteristics of PS are angiogenic, antinociceptive, gastroprotective, cardioprotective, etc. Their most common biomedical scope in medicines are wound healing, mucobioadhesion of bone and tissue, biolubrication in stiff joints, immunotherapy cancer vaccines, or as new versions of biotextiles and therapeutic fibres especially in drug delivery as well as promising platforms for regenerative remedy. For instance, *Porphyridium* and *Enteromorpha* PS have been confirmed as potent candidate for immunomodulation and antitumor possessions [141, 142]; PS from *Dyctiota menstrualis* and *Caulerpa cupressoides* are decent antinociceptive mediators [143, 144], whereas *Cladosiphon okaramanus* PS showed angiogenic, gastro- and cardioprotective properties [145, 146]. Table 5 demonstrating various specific microalgae prebiotic under nutraceutics, therapeutics and cosmeceutics applications. In which, the cosmeceutics is not a major focus of this article as it is a non-ingested for health application in GIT but implies on host skin surface.

## Challenges for commercialization and research advancement

With numerous bioactive compounds in algal biomass in which some are already arrived in commercial forms and playing an important role for human and animal health as functional food and animal feed. Consumption of these commercial forms already proved for positive health effects for various minor and major health issues. Still, scale up remains a major challenge for new compounds having prebiotic potential but yet to be tested at all stages before attaining commercial affordability [147]. Nevertheless, these obstacles, some probiotic companies have already overcome existing market constraints, and they are magnificently trading extracts and powder of microalgae as food supplement, colorant, and animal feeds. Solid technical evidence for probiotic roles of macro- and microalgae in humans and animals is awaited, while rare studies have addressed about delivering probiotic efficacy in marine animals upon these prebiotics ingestion [16].

Majority of the technological advancement in algal research failed to reach commercial stage mainly due to number of constraints. Primary is small market size; then production at higher cost than fossil materials. Others reasons are from chemical and biological routes covering fungal and bacterial process. Moreover, stringent regulations for safety assurance, quality specifications, and environmental impact reduction are also accountable [148]. Moreover, limitations in algal biomass harvesting and short viability are indeed constrained commercial success. Still, there is dispersed evidence about prebiotic potential of microalgae owing to their abundance in oligosaccharides which are barely fermented by GIT microbiota. Though, reliable applications occur only in the aquaculture and dairy industries. Commercial microalgal production facilities are scattered globally (Table 6) However, the majority of the facilities are dominated from North America and Asia, and rather less contribution by Europe, Africa, and the rest of the world.

**Table 6:** Commercial algal prebiotics products, their forms globally available for human health benefits upon consumption

Product name	Form of the product and application	Microalgae	Company	Production size (ton/year)	Reference
Spirulina Natural, Spirulina Gold	Tablets, powders, extracts	*Spirulina (world largest farm)*	Earthrise Nutritionals, California, USA	2000	[149]
Hawaiian Spirulina	Tablets, powders	*Spirulina pacifica*	Nutrex-Hawaii, USA	3000	[150]
Chlorella premium, Green gems, Chlorella Plus Chlorella Spirulina Tablets etc	Tablets, powders, nectar, noodles	*Chlorella sp.* *Spirulina sp.*	Taiwan Chlorella Manufacturing Company (TCMC) 1964	2000	[151, 152],
Chlorella supplements	Powders, tablets, extracts, drinks	*Chlorella sp.*	Hainan Simai Pharmacy Co. (China)	2000	[153]
Vitamineral Green	Powders, tablets, extracts	*Spirulina Azteca*	Health Force Nutritionals, Chile	–	[154]
FEBICO SOROKINA®	Powders (vitamins, proteins, dietary fibre, growth factors, phytochemicals etc.)	*Chlorella sorokiniana, Schizochytrium sp,*	Far East Bio-Tec Co., Ltd. FEBICO (ALGAPHARMA BIOTECH CORP.) Taiwan 1976	2000	[155]
JUNE Spirulina, SPIRUJU, Spilova wine, Juno fried chips etc	Tablets, extract, liquid chips, noodles and pasta	*Spirulina sp*	Myanmar Spirulina Factory	3000	[156]
ALGOMED® Chlorella natürlich	Powders	*Chlorella sp.*	Klotze (Germany)	2000	[156]
Hawaiian BioAstin	Tablets, powders,	*Haematococcus*	Nutrex-Hawaii, USA	3000	[150]
Hawaiian Spirulina and astaxanthin	Tablets, powders, beverages, extracts	*Spirulina sp., Haematococcus*	Cyanotech Corp. (USA)	3000	[150]
Blue green foods, Stem naturals, AFA organic dietary supplements	Capsules, crystal Powders, capsules	*Aphanizomenon flos-aquae*	Blue Green Foods (USA), Vision (USA)	500	[156]
Betatene®	Powders of β-carotene	*Dunaliella salina*	Cognis Nutrition and health (Australia)	1200	[156]
Astapure®	astaxanthin Powder in cosmetics	*Haematococcus pluvialis*	Algatech (Israel)	2000	[157]
AlgaVia™	Powder for flour supplement	*Chlorella sp.*	Solazyme	–	[158]

Regardless of the fundamental development in properties and functional food and animal feed formulation, wider research and development are prerequisite before macro- and microalgae are developed as a commercial realism in prebiotic formulation for several health applications. Table 6 summarizes various commercial microalgal products, compound forms, their brand names and manufacturing companies along with the production scales.

## Conclusion

Algae showed a marked potential to accomplish the people's alimentary and remedial needs, hence offer sustainable diet solutions. Coming years, the possibility of the potential use of algal prebiotics to regulate the gut microbiome specially to prevent several host diseases is anticipated. Besides being a rich source of amino acid, it’s potential for several bioactive compounds offers great promise for broader health applications. The opulence of nutritive as well as therapeutic compounds in microalgae provide a platform to raise an industry aimed to provide algae-based innovative functional foods which can boost not only nutrition scope of the host but also prophylactic effects. Currently, algal products are not affordable due to technological non-readiness as well as budget of scale in up- and downstream processes. Some obstacles need to be removed to launch the algae as a sustainable food solution for the rising population. Moreover, the prebiotic applications benefits offered by marine seaweeds and microalgae must not be limited to their PSs and lignin, but it must be rather wide up to other fractions such as PUFAs), monosaccharides, polyphenols, enzymes, alcohols, and peptides as these have been proved in analogous fractions of other sources. In the coming years, the likelihood of marine seaweeds PS as prebiotics, to modify the microbiome, and to get numerous health benefits is anticipated.

## Data Availability

Not applicable.

## Abbreviations

PS: Polysaccharide
HC: Hemicellulose
LAB: Lactic acid bacteria
DHA: Docosahexaenoic acid
EPA: Eicosapentaenoic acid
FOS: Fructo-oligosaccharides
XOS: Xylo-oligosaccharides
HMO: Human milk oligosaccharides
GOS: Galacto-oligosaccharides
SOS: Soya-oligosaccharides
LS: Lactosucrose
IMO: Isomalto-oligosaccharides
POS: Pectic-oligosaccharides
SCFAs: Short chain fatty acids
PDX: Polydextrose
IBS: Inflammatory bowel disease
IBD: Inflammatory bowel syndrome
ALGOS: Alginate-oligosaccharides
NAOS: Neoagaros-oligosaccharides
GLA: γ-Linolenic acid
PUFAS: Polyunsaturated fatty acids
